# Exorbitant Drug Loading of Metformin and Sitagliptin in Mucoadhesive Buccal Tablet: In Vitro and In Vivo Characterization in Healthy Volunteers

**DOI:** 10.3390/ph15060686

**Published:** 2022-05-30

**Authors:** Rouheena Shakir, Sana Hanif, Ahmad Salawi, Rabia Arshad, Rai Muhammad Sarfraz, Muhammad Irfan, Syed Atif Raza, Kashif Barkat, Fahad Y. Sabei, Yosif Almoshari, Meshal Alshamrani, Muhammad Ali Syed

**Affiliations:** 1 Department of Pharmaceutics, Faculty of Pharmacy, The University of Lahore, Lahore 54000, Pakistan; rshak123@hotmail.com (R.S.); rabia.arshad@bs.qau.edu.pk (R.A.); kashif.barkat@pharm.uol.edu.pk (K.B.); 2 College of Pharmacy, University of Sargodha, Sargodha 40100, Pakistan; sarfrazrai85@yahoo.com; 3 Department of Pharmaceutics, College of Pharmacy, Jazan University, Jazan 45142, Saudi Arabia; asalawi@jazanu.edu.sa (A.S.); fsabei@jazanu.edu.sa (F.Y.S.); yalmoshari@jazanu.edu.sa (Y.A.); malshamrani@jazanu.edu.sa (M.A.); 4 Department of Pharmaceutics, Faculty of Pharmaceutical Sciences, Government College University Faisalabad, Faisalabad 38000, Pakistan; 5 Department of Pharmaceutics, Punjab University College of Pharmacy, University of The Punjab, Lahore 54590, Pakistan; raza.pharmacy@pu.edu.pk

**Keywords:** mucoadhesion, antidiabetic, volunteer study, Carbopol^®^ 940, PVP

## Abstract

The aim of the proposed study is to develop a mucoadhesive buccal delivery system for the sustained delivery of metformin (MET) and sitagliptin (SIT) against diabetes mellitus (DM) with improved bioavailability. Polymeric blend of Carbopol^®^ 940 (CP), agarose (AG) or polyvinylpyrrolidone K30 (PVP) as mucoadhesive agents in formulations (R1–R15) were compressed via the direct compression technique. Tablets were characterized for solid state studies, physicochemical and in vivo mucoadhesion studies in healthy volunteers. Outcomes did not reveal any unusual peak or interaction between the drugs and polymers in the physical mixture through Fourier Transform Infrared Spectroscopy (FTIR) and DSC analysis. The mucoadhesive blend of CP and PVP was superior compared to other blends. The formulation R4 revealed exorbitant loading of drugs with complete drug release for 6 h with ex vivo mucoadhesive strength and time of 26.99 g and 8.1 h, respectively. It was further scrutinized to evaluate it as an optimized formulation where it was found to be stable for up to 6 months. The formulation R4 depicted Korsmeyer–Peppas model and first-order mode of release correspondingly for SIT and MET. Moreover, it showed hemocompatibility, biocompatibility and stability with non-significant changes in the dissolution profile. Overall, the CP blend with PVP was found appropriate to yield the desired release coupled with the optimized mucoadhesive properties of the buccal tablets, ensuring sufficient pharmaceutical stability.

## 1. Introduction

Diabetes mellitus type-II (DM) is perhaps the most potentially chronic disease prevailing worldwide, affecting quality of life with a greater incidence of clinical complications and mortality. The annual cost of treatment of DM complications accounts for approximately USD 500 billion. It can be treated properly with persistent glycemic control within normal ranges (70–140 mg per dL). Furthermore, the propagation of the manifestations of DM within the body are a major risk, which, if left untreated, chronic hyperglycemic concentrations can lead to retinopathy, hyperlipidemia, nerve degeneration and raised infection susceptibility [1,2,3]. Moreover, the varied concentrations of drugs in the blood or poor bioavailability may lead to the worsening of the clinical situation due to disease [4]. Therefore, the sustained delivery of anti-diabetic agents is of utmost need, while regulating the glucose concentration in blood [4]. Metformin, which is a biguanide agent, is currently considered as the mainstream option to treat DM. It exerts its pharmacological action by reducing basal and postprandial blood glucose [5]. Although metformin controls the hyperglycemic conditions in patients well, the combination of metformin and sitagliptin is to date the sole combination that is declared advantageous in terms of no weight gain and improving hypoglycemic situation [6,7,8]. However, conventional dosage forms lead to uncontrolled drug release, instability and less bioavailability of metformin [9]. Therefore, we addressed these concerns in our research via the formulation of buccal mucoadhesive tablets to ensure sustained systemic drug release with a low frequency of dosing [10]. Buccal mucoadhesive drug delivery has attained considerable interest in delivering local as well as systemic drug release [11,12] via the localization of the dosage form to the buccal cavity for drug absorption [11,13]. Moreover, localized intra-pocket, retentive, biodegradable, prolonged release buccal mucoadhesive tablets can provide an improved therapeutic efficacy of doxycycline at the site of action while evading off target side effects [10]. The combination of metformin (MET) and sitagliptin (ST) in this mucoadhesive system leads to patient compliance and maintaining control over levels of glucose [14].

As a result, the absorption and subsequent bioavailability of drugs are improved, leading to minimizing dosage intervals, which is correlated with a better patient compliance [11,15,16]. The loading of MET in orally sustained-release matrix tablets using a continuous melt granulation technique was achieved, ensuring biocompatibility and hemocompatibility [15,16,17]. Therefore, the aim of this study is to formulate mucoadhesive sustained-release buccal tablets with mucoadhesive polymers, such as CP and PVP [18], for a better drug loading of the candidate drugs. In this paper, we reported high drug loading into tablets with the least polymer concentration to sustain the release of the drugs. The impact of the polymeric blend on the mucoadhesion, strength, biocompatibility, hemocompatibility and release profile was investigated in vitro. Moreover, the in vivo bioavailability improved results clearly indicate the superiority of the novel synthesized mucoadhesive tablets.

## 2. Results and Discussion

### 2.1. Solid-State Characterization

#### 2.1.1. FTIR

**Figure 1:**
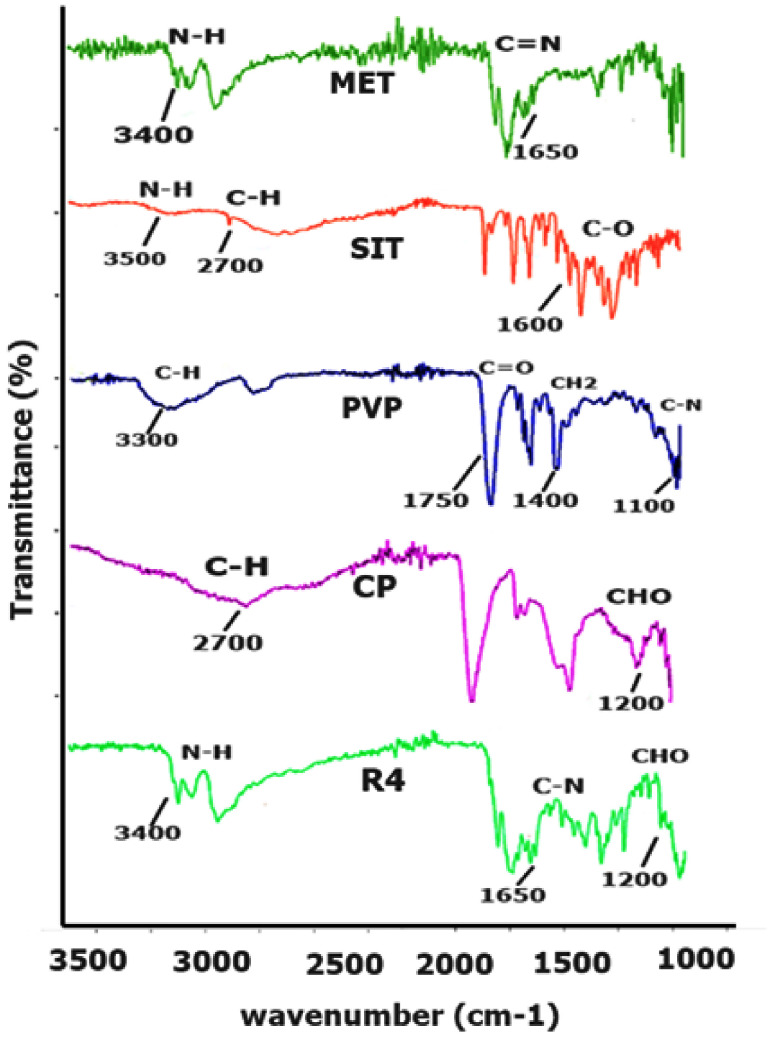
FTIR spectra of drugs, polymers and the physical mixture of drugs and polymers (in the ratio of the optimized formulation, R4).

The infrared peaks of pure MET, SIT, polymers (CP, AG and PVP) and physical mixture are presented in Figure 1. MET expressed a strong absorption band at 1650–1550 cm^−1^ due to presence of a C=N stretching vibration [19], while a weak C-N stretching of aliphatic diamine in the region of 1220–1020 cm^−1^ was observed. Similarly, the N-H stretching of the primary amine group occurred at 3400–3100 cm^−1^ and the N-H bending of the primary amine group occurred at 1640–1550 cm^−1^ (Figure 1. The FTIR spectrum of individual SIT showed significant bands at 3500–3300 cm^−1^, 3300–2700 cm^−1^ and 1780–1650 cm^−1^ that correspond to N-H, aromatic C-H and C=O stretching, respectively [20]. A predominant stretching of -OH in Carbopol was observed at 3300–2500 cm^−1^, 1780–1650 cm^−1^ (C=O of carbonyl group), 3300–2700 cm^−1^ (C-H bond), 1450–1375 cm^−1^ (C-H of CH_2_ bending) and 1300–1100 cm^−1^ due to asymmetric stretching of C-O-H [21]. Similarly, the corresponding characteristics peaks of PVP were observed at 1350–1000 cm^−1^ (C≡N stretching bands), 1780–1650 cm^−1^ (C=O of carbonyl group), at 3300–2700 cm^−1^ (C-H bond) and at 1465 cm^−1^ for the bending of C-H of CH_2_ [22]. Thus, the characteristic peaks of the ingredients were present in the physical mixture of the optimized formulation, and the absence of unusual peaks was confirmed.

#### 2.1.2. DSC

**Figure 2:**
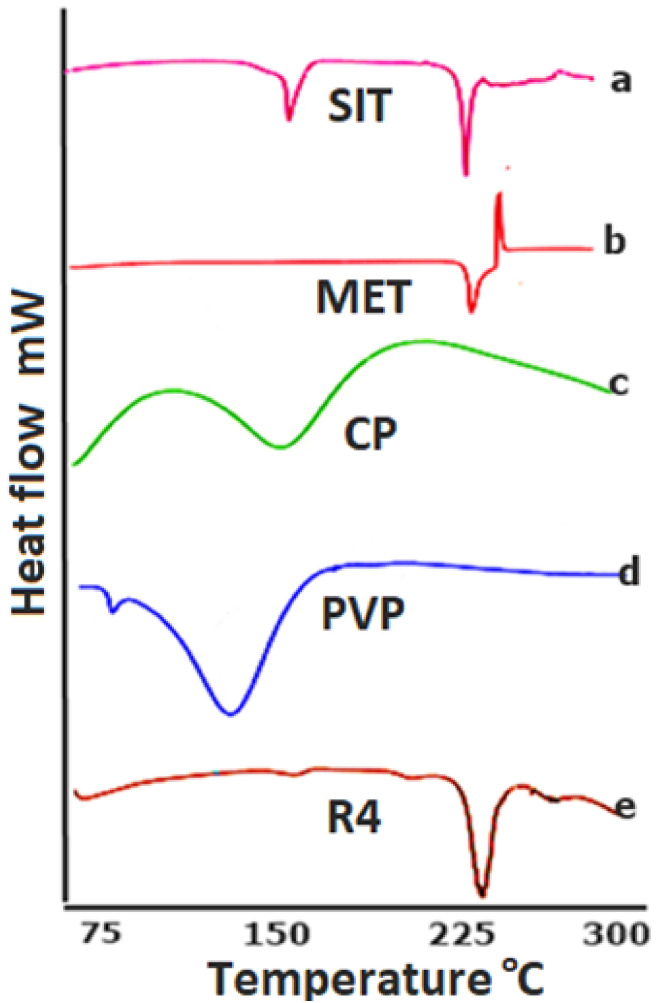
DSC thermogram of (**a**) sitagliptin phosphate monohydrate, (**b**) metformin hydrochloride, (**c**) Carbopol 940, (**d**) Polyvinyl pyrrolidone k30 and the (**e**) optimized formulation R4.

The DSC graph depicts the typical endotherm for CP corresponding with the literature. It depicts an endothermic change (Figure 2) around 113.62 °C as previously reported [23], while the endothermic curve of PVP was found to be similar as what has been previously reported [24]. Similarly, the endothermic changes in the peaks of SIT and MET could also be observed at the approximate values of 215.11 and 225.2 °C (Figure 2), respectively [20,25]. The endothermic curve of the physical mixture according to the optimized formulation reveals sharp peaks at the point of both drugs, indicating that the crystalline structures of SIT and MET were preserved in the compressed form. However, the endothermic changes in the physical mixture of the optimized formulation (in a ratio according to R4) demonstrate the peak at the point of drug is probably due to the higher and lower amounts of the drugs and polymer, respectively, in the formulation (Figure 2e).

### 2.2. Physical Characterization of Mucoadhesive Buccal Tablets

**Figure 3:**
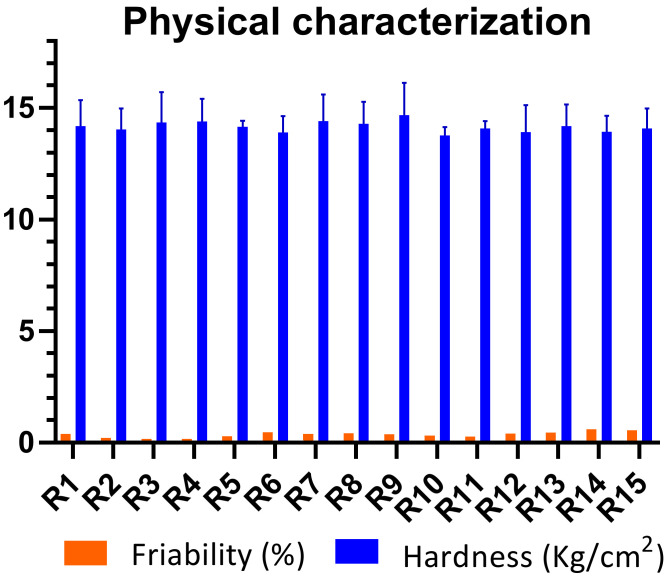
Hardness and friability of buccal tablets.

All the batches of buccal mucoadhesive formulations (R1–R15), containing different polymeric blends, were evaluated on various physical parameters (Table 1). According to the United States Pharmacopoeia (USP), a 5% deviation was allowed with a designated tablet weight of 650 mg for each formulation [26]. The average weight tablet formulation from R1 to R15 had values ranging from 648.2 ± 2.18 mg to 651.6 ± 2.13 mg. However, the designated deviations in all the formulations were below 5%, which shows that the weight variation complied with UPS limits. Hardness was, however, preset in the range of 13–15 kg/cm^2^ and so it was found to be within the designated range. The minimum hardness was reported for R6, which was 13.89 ± 0.71 (Figure 3), whereas the maximum hardness was found in R4 (14.39 ± 0.66). Likewise, the diameter and thickness of all batches (R1–R15) displayed minimum deviation. The diameter and thickness of all the formulations were within the range of 12.11–12.15 mm and 5.63–5.67 mm, respectively.

Moreover, the friability loss of the buccal formulations was less than 1%, indicating compliance with USP. Maximum friability was seen in the case of R14 (0.611%), while minimum friability was observed in the case of R11 (0.278%). 

It was observed that all batches of different formulations (R1–R15), despite having variations in the polymeric concentration, showed acceptable physical characteristics. The physical appearance of the prepared formulations, on the other hand, was also smooth, plain and with no pitted marks or abrasions. In conclusion, the changing concentrations of polymeric blends used in the study did not cause significant variations on the physical parameters in terms of non-compliance with compendia specifications.

### 2.3. Physicochemical Characterization

#### 2.3.1. Content Uniformity

A content uniformity test was performed to ensure that the amount of active ingredients was present in full concentration as stated. The results for MET and SIT reveal that the percentage of drug content in all batches was in between 97.08 ± 1.29 and 102.40 ± 0.95 (for MET) and 97.13 ± 0.78 and 102.71 ± 0.98% (for SIT), respectively (Table 2). The unit contents of active substances should be in the range of 95–105%, according to United States Pharmacopeial (USP) standards. The results of both SIT and MET complied with USP specifications.

#### 2.3.2. Surface pH

Surface pH is a crucial parameter regarding the adjustment and lodging of the buccal dosage form in the buccal environment since drastic pH values may irritate buccal mucosa. It can, in turn, cause redness, inflammation or worsen the clinical situation. Moreover, the polymer also works on a narrow range of pH to show its pharmaceutical properties [27]. The pH of the prepared formulations was found to be in the range of 4.74–7.11 (Table 2). The maximum pH value was observed in R1 (Table 2), which was 7.11, while low pH was seen in R15 (4.74). There have been different studies on the opinion of normal pH for the mucosa. A study reported that a pH range from 5.5 to 7.0 is considered suitable with respect to the buccal environment [28], while on the other hand, salivary pH in the range of 6.2–7.6 is also considered as normal [29]. Considering these findings, it is clear that formulations R11–R15 containing a polymeric blend of AG and CP exhibited comparatively lower surface pH values than the rest of the formulations.

#### 2.3.3. Swelling Index

**Figure 4:**
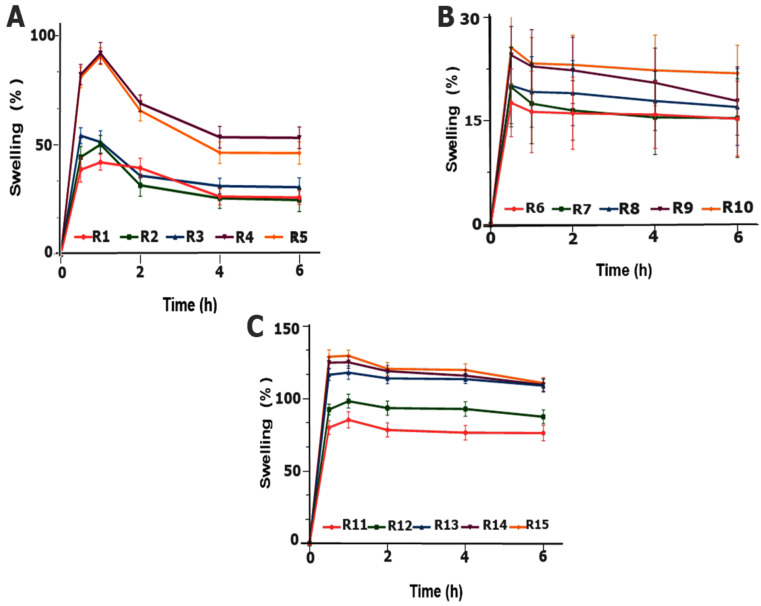
Swelling index of the formulations: (**A**) R1–R5, (**B**) R6–R10 and (**C**) R11–R15.

The swelling study indicates the capacity of the polymer to imbibe water for swelling, thereby producing mucosal adherence and ultimately controlling the release of drugs over time [30]. Among all polymeric blends, the maximum swelling trend was found in the blend R15 (CP and AG), which was 129.71% (Figure 4A) at 1 h, containing a maximum amount of CP and a minimum amount of AG of 12% and 2%, respectively. The findings support the mucoadhesive findings of AG and CP tablet delivery where the combination of AG and CP improved the swelling as well as adhesive properties of the dosage form [31]. The carboxyl group in carbopol dissociates at pH 6.8 as a result it forms a thick swollen gel, displays an excellent gel forming ability [32]. As far as swelling is concerned, it is assumed that, when CP comes in contact with water, it transforms from tightly closed interlocking chains to an uncoiled structure, leading to the revelation of its carboxylic group [33] and an electrostatic repulsion between negatively charged carboxylic groups causing the molecule to expand; ultimately, swelling and gelling occur and the resultant gel contains closely packed swollen particles. Similarly, in the case of R15, which exhibited maximum swelling, the gel forming property was attributed to CP and an increased concentration was associated with increased values of SI. 

In formulations F6–F10 (Figure 4B), the polymeric blend showed poor swelling and the highest swelling was 25.6% in R10 (AG: PVP, 12: 2%) after half an hour. The formulations start eroding after an hour. Therefore, AG in the tablets showed poor hydrophilic interaction with the media in the experimental conditions. Consequently, poor swelling was associated with the stated formulations [34]. 

**Figure 5:**
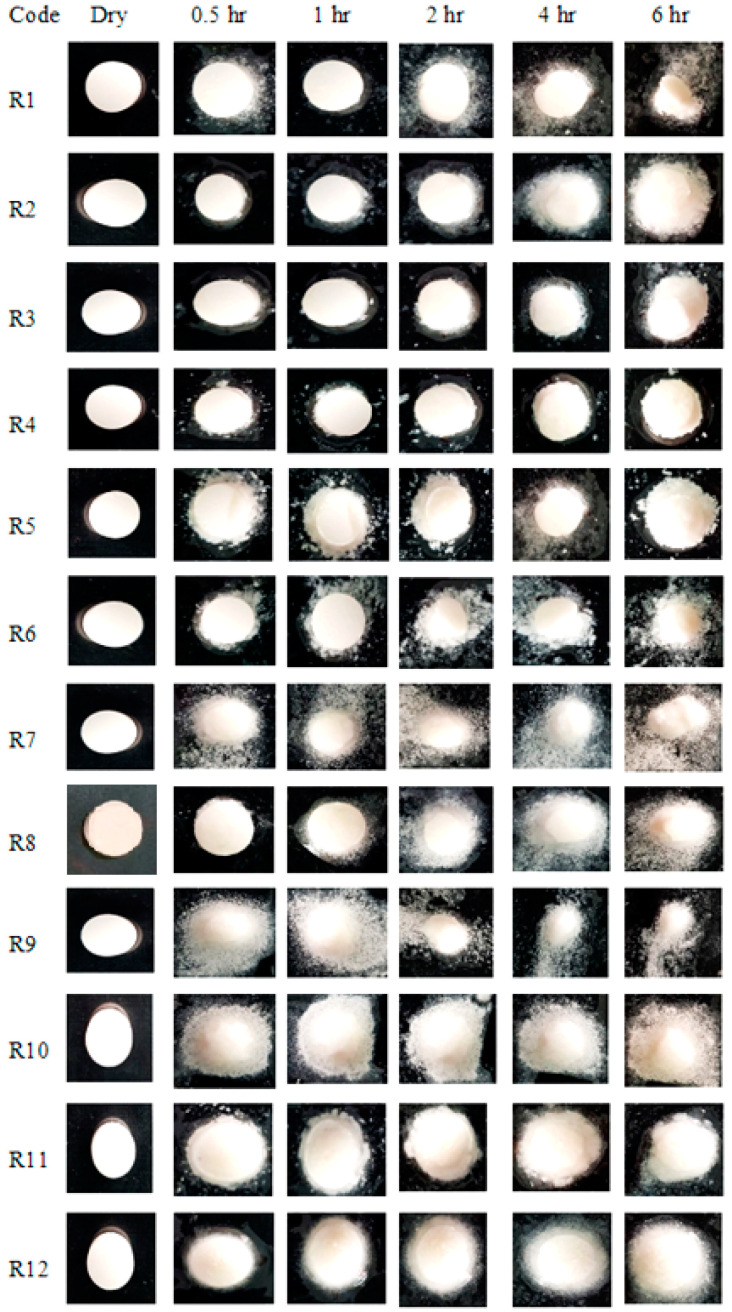
Photographic swelling pattern of the buccal formulations.

While in formulations R1–R5 (Figure 5) swelling occurred as a function of the concentration of CP in the formulation, the formulation R4 displayed the highest swelling of 91.9% at 1 h. This could be attributed to the better hydration of polymers as both CP and PVP are hydrophilic and have the ability to imbibe water fast. As the concentration of CP increased, SI correspondingly increased, probably due to the hydration of the polymer via the subsequent ionization of the carboxylic group [35]. It was assumed that the addition of the hydrophilic polymer PVP with CP increased the surface hydration and water penetration within the matrix; thus, fast swelling occurred within one hour. Additionally, the physical appearances of the swollen tablets of all formulations (R1–R15) at specified intervals were also captured photographically (Figure 5).

#### 2.3.4. Matrix Erosion

In the formulations R1–R5, erosion was higher (Table 2) with a greater amount of PVP and the tablet lost physical integrity, whereas, it was comparatively lower with lower concentrations of CP (Table 2). This might be due to the hydrophilic nature of the solubilized PVP when in contact with the media, while the adhesive nature of CP reduced its erosion. In the formulations (R6–R10) that had AG and PVP, tablets depicted (Table 2) poor swelling with a higher degree of erosion. The polymeric blend was not able to retain the swollen tablet integrity and it dispersed and eroded after 0.5 h presumably due to the poor hydrophilic interaction of AG in water under the experimental conditions [31]. The incorporation of AG, even at a high concentration (% *w*/*w*), did not provoke reasonable swelling and the tablets started to erode rather than swell. Furthermore, the blend of a water-soluble polymer (PVP) might cause the migration of PVP during drying. Erosion might be due to either a lower drug to polymer ratio or the hydrophilic nature of both the polymers and drugs. While formulations R11–R15 exhibited (Table 2) a lower erosion as compared to the other groups of formulations, the lowest erosion and the highest swelling were related with the concentrations of CP, which could possibly be due to the higher concentrations of CP.

#### 2.3.5. Ex Vivo Mucoadhesive Time (ET)

**Figure 6:**
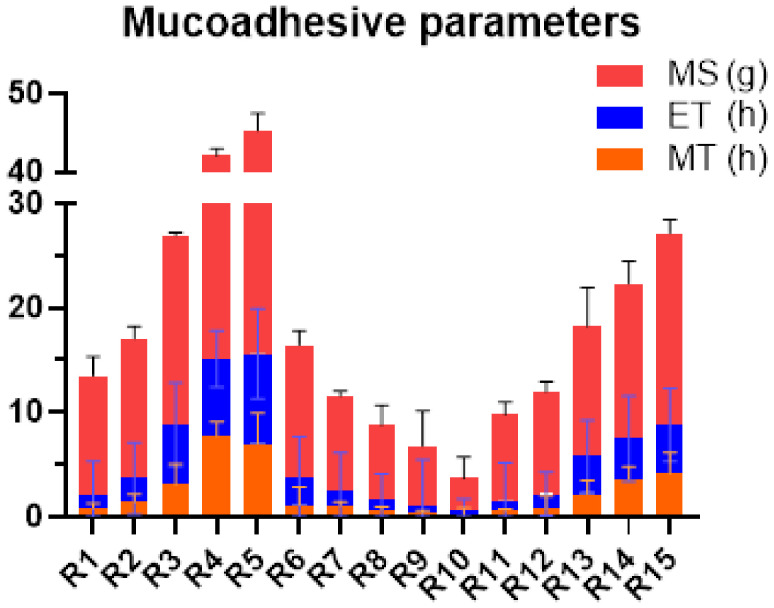
Comparison of the mucoadhesive parameters of the fabricated buccal formulations.

The results depict that the formulations R4 and R5 possessed a higher ET, which were 7.38 and 8.60 h, respectively. It was again related with the concentration of CP and its mucoadhesive capabilities [32] in the presence of a hydrophilic polymer (PVP) [36]. Instead, the formulations containing AG and PVP (R6–R10) presented the lowest ET values. However, the present study did not reveal the synergistic effects of PVP for swelling and mucoadhesion with the addition of PVP. In the formulations R11–R15 (AG and CP), the mucoadhesive character of CP might have resulted in the swelling of R15 in addition to a considerable residence time of 1.5 h (Figure 6). The incorporation of AG in the tablet dosage form did not yield significant results as it promoted the sudden dispersion of the tablet upon contact with water [31].

Symptoms of redness, inflammation or any irritation were not found from the volunteers, which suggests that the drug delivery was adaptable in the buccal mucosa without producing local irritation or pain.

#### 2.3.6. Mucoadhesive Strength (MS)

The force required to detach the tablet from mucosal membrane is considered as mucoadhesive strength. Highest MS was observed with formulation R5 (29.68 g), compared to R10 having lowest value (Figure 6). CP contained good adhesive properties, while AG and PVP were less likely to show adequate adhesiveness. There was an overall increasing trend of MS associated with the concentration of carbopol. Upon hydration, the ionization of the carboxylic group in Carbopol with subsequent swelling established hydrogen bonds in the mucin layer. This formation of hydrogen bonds coupled with an increased degree of swelling was responsible for the strongest mucoadhesion [26]. Furthermore, it is reported that high wettability, as in the case of the water soluble polymers, results in the enhanced diffusion of polymeric chains into the mucus membrane in a short span of time, giving rise to excellent mucoadhesion strength [37]. The mucoadhesion of CP is related to the formation of an adhesive interaction in which the polymeric chain produces adhesion with mucus membrane. Thus, CP exhibited excellent MS properties and the least was observed in AG in the powdered form [38]. The results indicate (Figure 6) that the combination of CP with PVP, as in the case of R1–R5, showed the maximum mucoadhesion, while CP with agarose, as in the case of R11–R15, produced a lower mucoadhesive strength [31].

#### 2.3.7. Mucoadhesive Study in Volunteers (MT)

The outcomes of MT of better values of residence time in volunteers were found in R4 and R5, containing a blend of CP and PVP. The corresponding MT for the formulations was 7.73 and 6.85 h. For such formulations, the tablet remained adhered to the mucosa and did not erode. Low values of MT were observed in the formulations R6–R10 comprising AG and PVP. The formulations did not last for more than an hour at the site of application. However, the formulations R11–R15 (AG and CP) possessed moderate values for MT. 

#### 2.3.8. In Vitro Hemolytic Analysis

**Figure 7:**
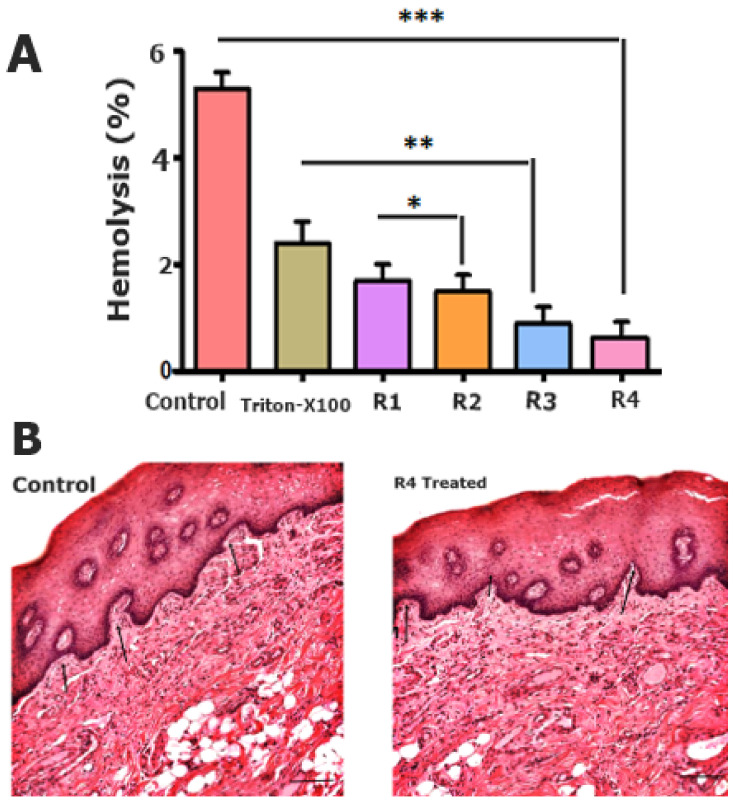
In vitro hemolytic (%) assay (**A**). The p value less than 0.1, 0.01 and 0.001 were denoted with *, ** and *** respectively; and histopathological evaluation of the buccal mucosa (**B**).

Hemocompatibility is an essential parameter for determining polymeric excipient compatibility with the drug formulation [39]. Mucoadhesive tablets hemolysis% ranged in the safe zone, midway of 2–4% as shown in Figure 7A, whereas the optimized formulation R4 showed hemolysis of only up to 0.2%. The reduced hemolysis of R4 is due to the biocompatible combination of polymeric excipients in mucoadhesive tablets.

#### 2.3.9. In Vivo Histopathological Evaluation

A histopathological evaluation of the buccal mucosa was performed to determine any changes or deformities after treating with the optimized R4 formulation, as shown in Figure 7B. Moreover, the mature epithelialization of the mucosal tissues was compared to that of the other formulations and the control group. However, it was evident that the R4 formulation possesses a strong biocompatibility and safety features, owing to the use of moderate ratios of optimized polymeric combinations. 

#### 2.3.10. In Vitro Cytotoxicity Analysis 

**Figure 8:**
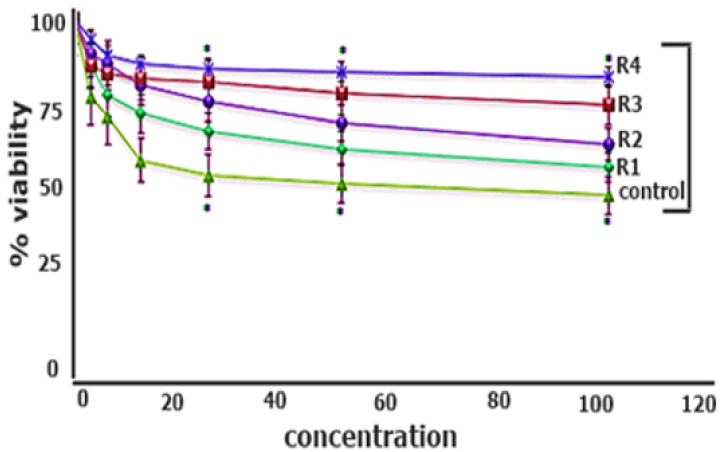
Biocompatibility assay of the mucoadhesive polymeric tablets. The results are listed as mean ± S.D (*n* = 3) where the * means a significance level of 0.001.

A biocompatibility assay was performed using a range of concentrations of mucoadhesive polymeric tablets. The results reveal the biocompatibility of the R4 optimized formulation _by_ showing a cell viability of 98% at a higher drug proportion. Similarly, in literature, it is evident that a polymeric combination results in increased biocompatibility [40]. However, the cell viability for all the formulations with a modified polymeric system was also higher than 70% as compared to the control, i.e., 45%, with a statistical significance of (*p* < 0.05), as shown in Figure 8.

#### 2.3.11. In Vitro Drug Release 

##### Analytical Quantification of Metformin (MET) and Sitagliptin (SIT)

The peaks of MET and SIT were separated according to the devised conditions with the respective values of 1.96 and 3.70 min, as reported in the preliminary findings associated with the current study [41]. 

##### Dissolution Study

**Figure 9:**
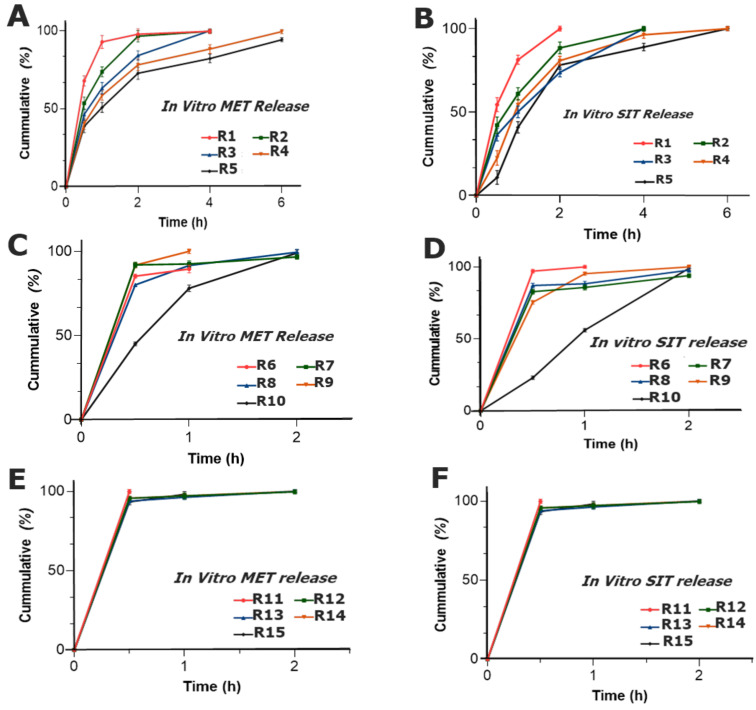
Cumulative release of (**A**) MET and (**B**) SIT of the formulations R1–R5; (**C**) MET and (**D**) SIT of the formulations R6–R10; and (**E**) MET and (**F**) SIT of the formulation R11–R15.

The change in the concentration of the polymers produced relative changes in the release profile of both drugs. In formulations R1–R5, the release behavior showed that, by increasing the concentration of CP, the release of both drugs decreased and had a sustained effect within 6 h of the study. This could be due to the strong gel matrix that was formed by CP that eventually retarded the release pattern of the drugs; thus, as a result, the cumulative drug release decreased [42]. It was also reported that CP above concentrations of 5 to 30% yields a brilliant release retardant property to a dosage form [43]. Furthermore, the binding effect of PVP imparted an additional release retardant effect in the presence of CP and has shown an effective holding capacity for both water soluble drugs, whereas R6–R10 (AG and PVP) expressed a burst release of both MET (Figure 9C) and SIT (Figure 9D) and the drugs were completely released within 2 h of the study. AG did not show a considerable uniform sustained release pattern in the tablet dosage form. The results depict that, at such a ratio of polymers of AG, sustainability in drug release was not achieved and showed a poor release retardant effect. This might be due to the fact that AG possesses weak hydrophilic forces in powder form [44], while PVP, the hydrophilic polymer, did not impart the required results with the release of both drugs, and the tablets diminished completely after 2 h of sampling. Consequently, the drugs dissolute with a faster release from eroded matrix [45]. 

**Figure 10:**
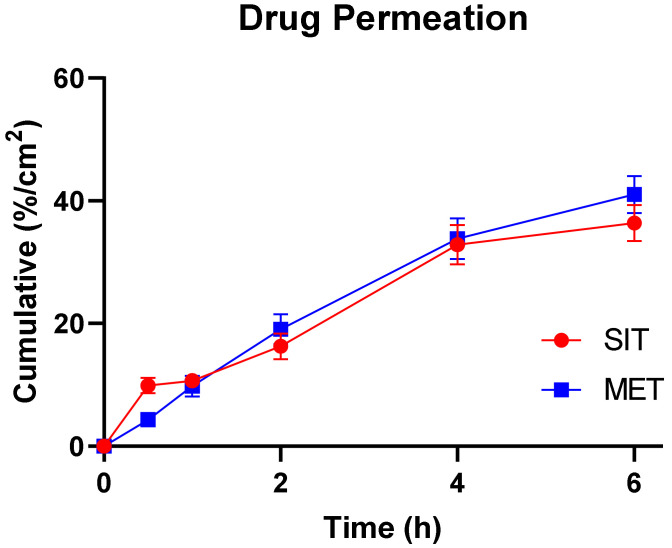
Cumulative percentage of drugs permeated through the unit area of rabbit buccal mucosa from the optimized formulation.

Similarly, R11–R15 (CP and AG) exhibited a burst release of MET (Figure 10E) and SIT (Figure 10F), as more than 80% of both drugs were released within 0.5 h of the study. The combination of CP with AG failed to yield the release retardant properties in the formulations R11–R15. Even with the increase in CP, from R11 to R15, the release was sustained, but not up to the required extent, whereas increments in AG were also unable to produce the sustained release. 

### 2.4. Optimization of Formulation

Based on the complete in vitro drug release and optimal mucoadhesive properties, the formulation R4 was selected, since it exhibited the complete release of MET (>99%) and SIT (100%) until 6 h with considerable mucoadhesive properties as well. Moreover, R4 also possessed a better mucoadhesive strength of about 26.99 g (0.265 N) with a residence time of about 2.5 h and the swelling trend showed 53.2% of hydration. Therefore, R4 was further evaluated for ex vivo drug permeation, stability study and solid-state characterization.

#### 2.4.1. Ex Vivo Permeation Study

The permeation flux of both drugs increased over time until 6 h (Figure 10). Regarding R4, it was assumed that drug molecules diffused passively from the mucosal membrane since it is considered to be the main mechanism of absorption for both drugs [46]. Sitagliptin and metformin exhibited a flux of 13.85 and 7.35 mg/cm^2^.h (Table 3). The flux was not found to be saturated at its highest donor concentration, which conforms to the hypothesis that both drugs actually permeate through passive diffusion. However, the amount of SIT and MET did not completely permeate until the end of 6 h (Figure 10). 

#### 2.4.2. In Vitro Release Kinetics

Drug release kinetic models were applied using DD solver^®^ and the best model was selected on the basis of the maximum value of the coefficient of the drug release kinetic model (r^2^). In the case of MET, the maximum value of r^2^ was calculated for the Korsmeyer–Peppas model compared to rest of the models and the value was found to be 0.9633 (*n* = 0.321). This means that the dissolution of water-soluble drug out of the dosage form was driven by non-Fickian-based diffusion only, since the value of *n* was lower than 0.45, as shown in Table 4 [47]. This shows that the release of drugs from the swollen tablet was based after the formation of the gel (peripheral swollen tablet) around the solid tablet core from which MET diffused (Figure 6). 

Unlikely to MET, the best fit model to describe the in vitro release kinetics of SIT was the first-order model as it depicted a maximum coefficient value (r^2^ = 0.9765), compared with others (Table 4). It meant that the dissolution of SIT out of the dosage form was based on a concentration-based mechanism. So, initially, when the concentration was at a maximum, the release of SIT was high and it proportionally decreased with the concentration over time.

#### 2.4.3. Stability Study

The testing of the optimized formulation under standard regional conditions was performed for physical appearance, ET, MS and the total drug contents in the formulation at specified intervals until 6 months. It was found that the total drug contents in the formulation were almost higher than 98% until 6 months (Table 5). This demonstrates that the solid contents of the formulation were safely retained during stressful conditions. The ET and MS values pertaining to R4 show negligible variation during the study period, which shows that the dosage form was stable under stability conditions.

#### 2.4.4. Statistical Analysis

Similarity (f1) and dissimilarity (f1) factors were also applied on the release profile of both drugs evaluated before and after stability. For f2, the values of SIT and MET were found to be 62.35 and 67.47, correspondingly, while for f2, the values were 7.17 and 5.88, respectively. These factors were found to be in accordance with the acceptable limit of the similarity (50–100) and dissimilarity (0–15) ranges [11], which conforms that the release profile of the formulation did not differ significantly for both SIT and MET (Table 6). Consequently, the solid form was considered stable during the storage time period under the specified conditions (stability zone IVa). The data were analyzed by employing a Student’s paired *t*-test to find significant differences between the paired samples of drug release before and after stability conditions (Table 7). As observed, the *p*-value was found to be greater than (*p* > 0.05), which meant that there was no significant difference between the release profile of the optimized formulations before and after the conditions of stability at a 95% confidence interval. The smaller value of t also demonstrated that an insignificant difference exists in the means of the release profile of both drugs.

## 3. Materials and Methods

### 3.1. Materials

Metformin hydrochloride (MET) and Carbopol 940^®^ (CP) were kindly provided by Hoover Pharmaceuticals. Pvt. Ltd. (Lahore, Pakistan), whereas Sitagliptin phosphate monohydrate (SIT) was collected from CCL Pharmaceuticals, Lahore, Pakistan. Agar extract (AG) with gel strength ≥1200 g/cm^2^, gelling temperature of 35–37 °C and melting point of 87–89 °C was purchased from bio WORLD^®^ (Dublin, OH, USA). Limited. Similarly, polyvinylpyrrolidone k30 (PVP), magnesium stearate and lactose were generously donated by Wilshire Laboratories Pvt. Limited, Lahore, Pakistan. Distilled water was used throughout the study unless otherwise stated. 

### 3.2. Formulation of Mucoadhesive Buccal Tablets

Fixed concentration additives as well as variable amounts of mucoadhesive polymers were weighed according to quantities mentioned in the master formula (Table 8). Then, the ingredients were mixed geometrically with the help of a mini pestle and mortar for 5 min or as required. Geometrical mixing was performed in such a way that the diluent was added last. This polymeric blend was shifted to an already lubricated die cavity of ZP-35 rotary tablet machine that was operated manually. It was then compressed into tablets by direct compression using an 8 mm flat faced punch by exerting a force of 2.5 tons for 10 s [45]. 

### 3.3. Dosage Form Design 

In this current study, fifteen different formulations (R1–R15), containing different polymeric blends of CP, PVP and AG, were directly compressed. Formulations were designed to evaluate the impact of the varied concentrations of polymers on physical as well as physicochemical parameters. All the prepared formulations were characterized for physical evaluation, surface pH, swelling index, matrix erosion and mucoadhesive characterization. Subsequently, dosage form was optimized based on optimum swelling, mucoadhesion and in vitro drug release till 6 h. The optimized formulation was then evaluated for in vitro drug release kinetics, stability studies, adaptability response of the volunteers, biocompatibility and hemocompatibility. The fixed dose combination of MET (500 mg) and SIT (50 mg) was used in all formulations. Similarly, a fixed 2% *w*/*w* concentration of magnesium stearate was used as a lubricant. All the polymers were evaluated at a concentration of 2, 4, 7, 10 and 12% *w*/*w* (Table 8).

### 3.4. Solid-State Characterization

#### 3.4.1. Fourier Transform Infrared Spectroscopy (FTIR) Analysis

FTIR analysis was carried out in order to investigate any unusual peak that could possibly depict any interaction between drugs and excipients. Concisely, powdered samples of pure drugs, polymers and optimized formulation and its respective physical mixture were analyzed using Bruker ALPHA^®^ (Bruker, Billerica, MA, USA) attached with OPUS^®^ Software (Alpharetta, GA, USA). The FTIR spectral scanning was carried in the range of 4000–600 cm^−^^1^.

#### 3.4.2. Differential Scanning Calorimetry (DSC)

The DSC analysis was performed on drugs, polymers and the physical mixture of the ingredients according to the composition of the optimized formulation using a differential scanning calorimeter DSC TL Q 2000^™^ (TA Instruments, New Castle, DE, USA). Approximately, 10 mg of each sample was placed inside the aluminum cup and then covered with the lid. It was then placed inside the heating chamber of the machine that scanned heat into the system over a range of 40–300 °C at an incremental speed of 20 °C/min. The gas was purged at a rate of 50 mL/min [11].

### 3.5. Physical Characterization of the Formualted Tablets

Formulations were evaluated for weight variation, diameter, thickness, hardness, friability and content uniformity [26]. The weight variation test was performed on twenty tablets from each code. These were subjected to be weighed individually and the average weight for each formulation was calculated to estimate the standard deviation. Thickness and diameter were calculated using digital Vernier caliper on the formulated tablets and the results were expressed in terms of standard deviation [12], while hardness was determined by using automated hardness tester Curio HT-901 on ten tablets. The friability was estimated by Roche friabilator^®^ (Scientific Supplies, Karachi, Pakistan) wherein approximately 6.6 g of sample was placed in a friabilator at 25 rpm for 4 min. Then, the tablets were dedusted and reweighed. Finally, friability was calculated (Equation (1)):

(1) $$\mathrm{Friability}\;\left(\%\right)=\frac{\mathrm{Initial}\;\mathrm{weight}-\mathrm{Final}\;\mathrm{weight}}{\mathrm{Initial}\;\mathrm{weight}}\;\times \;100$$

### 3.6. Physicochemical Characterization of Mucoadhesive Buccal Tablets

#### 3.6.1. Surface pH

For the measurement of surface pH, the tablets were allowed to swell in 10 mL of distilled water adjusted to pH 6.8 for 2 h, at 37.5 °C on a hot plate. The surface pH was then measured by touching the electrode of the pH meter on the surface of the tablet and allowing it to equilibrate approximately for 1 min [26,41]. 

#### 3.6.2. Content Uniformity

Briefly, three tablets from each formulation code were separately crushed in a pestle and mortar and the weight of the powder equivalent to the single tablet was taken. It was placed in a volumetric flask containing 900 mL of the dissolution media for the solubility of the drug. Then, it was stirred magnetically for 45 min at 1000 rpm and 5 mL of the fluid was filtered. Eventually, it was injected in the HPLC machine through an injector for analysis using the HPLC instrumental conditions as reported by the authors previously [48].

#### 3.6.3. Swelling Index (SI)

Initially, the tablets were weighed from each formulation code considered as W1. Each tablet was then kept in a Petri dish over a glass slide containing 10 mL of distilled water adjusted to pH 6.8 in such a way that half of the tablet remained immersed in the medium. After intervals of 0.5, 1, 2, 4 and 6 h, the tablets were withdrawn from the Petri dishes and the swollen tablets were reweighed (W2) in order to measure the weight gained by the respective tablet [11]. The degree of swelling was calculated as (Equation (2)). The experiment was performed in triplicate and the average values were expressed as standard deviation [45].

(2) $$\mathrm{SI}=\frac{W2-W1}{W1}\times 100$$

#### 3.6.4. Matrix Erosion

The swollen tablets at the end of time period of swelling index study (6 h) was placed in the dried air oven at 60 °C for 24 h in a drying oven to be dried for 48 h [11]. It was reweighed as W3. Then, the erosion of the tablet was calculated as follows (Equation (3)):

(3) $$\mathrm{Matrix}\;\mathrm{Erosion}=\frac{W1-W3}{W1}\times 100$$

#### 3.6.5. Ex Vivo Mucoadhesive Time (ET)

The ET was performed on excised buccal mucosa, which was fixed to a glass slide with an acrylate adhesive. Afterwards, one face of the tablet was wetted with approximately 500 µL of PBS and was then pressed gently for few seconds on the excised tissue to fix mucoadhesive tablet for ET evaluation [11]. Thereafter, glass slide containing attached tablet was immersed in the beaker at an angle of 45° containing 900 mL of phosphate-buffered solution (PBS), adjusted to pH 6.8 with phosphoric acid [12]. The temperature of the apparatus was maintained at 37 °C during the experiment with a stirring speed of 100 rpm. The time at which a tablet either detached or disintegrated from the mucosal surface was considered as ET.

#### 3.6.6. Ex Vivo Mucoadhesion Strength (MS)

**Figure 11:**
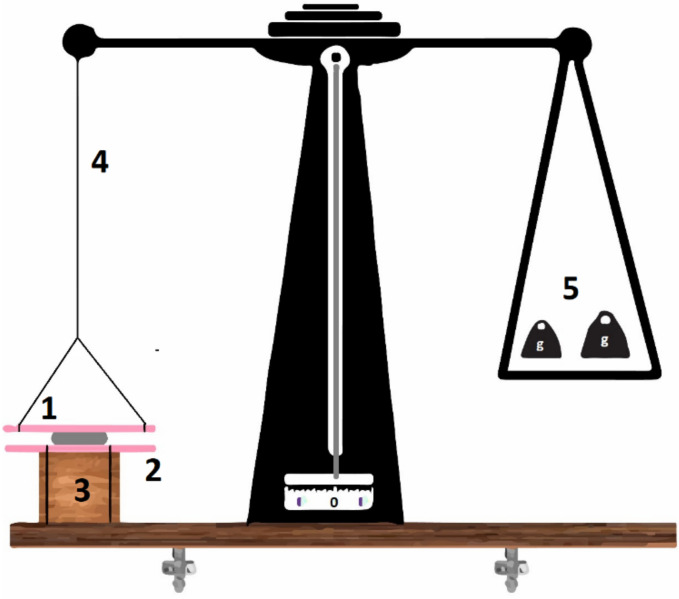
Experimental simulation of mucoadhesive strength tester as reported previously [31], where 1 = moveable glass slide with attached mucosa, 2 = attached mucosa to fixed glass slide to 3 = base, 4 = modified arm and 5 = weight added to the other arm.

The estimation of MS was carried out using a modified physical balance. Concisely, one arm of the scientific physical lab balance was replaced with thread and two glass slides, in between which the buccal tablet was placed (Figure 11). The fixed glass slide was attached to the base, while the moving glass slide was attached to the arm of the physical balance as reported [13,45]. Then, the surface of tablet was moistened with 250 µL of PBS and placed in between mucosal membranes. Both slides were pressed gently for a few seconds. When the system was stable, weight was added in the other pan. The mass (g) at which the tablets detached from the mucosa was considered as a mucoadhesive strength [12]. 

#### 3.6.7. Mucoadhesive Time in Volunteers (MT)

All protocols of the Declaration of Helsinki were followed. Moreover, the amounts of MET and SIT in all the formulations being tested for MT in volunteers were replaced with the diluent added as the experiment was not intended to determine the salivary or plasma concentration of drugs in subjects.

Briefly, six volunteers, who were instructed about the procedure of the study and willing to participate were included. The subjects did not have any acute or chronic forms of concurrent disease and were aged 20–28 years. Individuals were not permitted to consume solid food during the experiment except the gentle intake of a liquid diet. Initially, the mucoadhesive buccal tablet was applied gently on the frontal side of buccal mucosa beneath the parotid duct. The time was recorded at which the tablet either disintegrated, completely dissolved or detached from the point of application and was considered as MT. The incidence of pain, swelling or irritation perception was also recorded from the volunteers during the experiment [11].

#### 3.6.8. In Vitro Drug Release

##### HPLC Instrumental Conditions

High Performance Liquid Chromatographic (HPLC) method was adopted with instrumental conditions as reported by Aqeela et al. [41] for the simultaneous estimation of metformin hydrochloride and sitagliptin phosphate monohydrate in the mucoadhesive tablet. Briefly, the mobile phase was composed of acidified water: methanol (6:4) volumetrically and adjusted to pH 3.0. It passed through C_18_ column (250 × 4.6 mm, 5 μm), a maintained at 25 °C throughout the analysis and the detector was set to absorb ultraviolet radiations at 260 nm, meanwhile the mobile phase was flowing at a rate of 1 mL/min.

##### Dissolution Study

A dissolution study was conducted using USP type II apparatus to simulate in vivo conditions. To each vessel, a tablet was added in 900 mL of PBS, adjusted to pH 6.8. The dissolution media was maintained at 37.5 ± 0.5 °C with a paddle speed of 50 rpm [49]. Samples of 5 mL were withdrawn at a predetermined time interval (0.5–6 h) with the equal volume replenished with fresh medium. Samples were filtered with 0.2 μm syringe filter and analyzed for percent drug release by HPLC [50].

#### 3.6.9. In Vitro Drug Release Kinetics

A kinetic analysis was performed on the outcome of the dissolution study of the optimized formulation using DD solver^®^. Different kinetic models were applied on the cumulative release of MET and SIT from the optimized formulation, which included zero-order, first-order, Higuchi, Korsmeyer–Peppas and Hixson–Crowell models [11]. The mode of drug release was then studied based on the maximum value of the coefficient of kinetic models.

#### 3.6.10. Ex Vivo Permeation Study

The amount of SIT and MET that could permeate through the buccal mucosa from the optimized formulation was evaluated using buccal mucosal membranes of rabbits that were isolated carefully and preserved for instant use on Franz diffusion cells [30]. Briefly, the mucosa was held between the donor and receiver compartments with an active area of 1.1236 cm^2^ of the dosage form in contact with the membrane. The receiver compartment contained 9 mL of the simulated saliva at pH 6.8; meanwhile, the whole system was maintained at 37.5 ± 0.5 °C and stirred at 50 rpm. Then, one mL of salivary fluid was added to the donor compartment to mimic the physiological conditions of saliva in the donor compartment. At specified time intervals, aliquots of 1 mL were withdrawn from the sampling port and were analyzed using the devised HPLC technique. Steady state flux (J_ss_) and permeability coefficient (K_p_) were calculated by Equations (4) and (5), respectively [51]. The permeation study was carried out three times to evaluate the permeability coefficient.

(4) $$\mathrm{Steady}\;\mathrm{state}\;\mathrm{flux}\;\left(\mathrm{Jss}\right)=\frac{\mathrm{Slope}}{\mathrm{Area}}$$

(5) $$\mathrm{Permeability}\;\mathrm{coefficient}\;\left(\mathrm{Kp}\right)=\frac{\mathrm{Jss}}{\mathrm{Total}\;\mathrm{drug}}$$

where J_ss_ is steady state flux (mg/cm^2^.h) and K_p_ is permeability coefficient (cm/h).

#### 3.6.11. In Vitro Hemolytic Analysis

Hemolysis assay was performed via collecting 5 mL human blood samples from healthy volunteers (with their permission) in an anticoagulant tube. Fresh human blood was rinsed three times with a normal saline solution (0.9% *w*/*v* NaCl), followed by dilution with Dulbecco’s phosphate-buffered saline (DPBS) in a mixing ratio of 1:9. Furthermore, different concentrations of mucoadhesive tablet formulations (1–3 mg/mL) were added into the diluted blood followed by incubation at 37 °C. Furthermore, samples were centrifuged at 4500 rpm for 15 min at 4 °C for supernatants were collected and analyzed for absorbance via a micro titer plate reader at 540 nm [52]. Percent hemolysis was calculated using Equation (6).

(6) $$\%\;\mathrm{hemolysis}=\frac{\mathrm{absorbance}\;\mathrm{of}\;\mathrm{sample}-\mathrm{absorbance}\;\mathrm{of}\;\mathrm{negative}\;\mathrm{control}}{\mathrm{absorbance}\;\mathrm{of}\;\mathrm{positive}\;\mathrm{control}-\mathrm{absorbance}\;\mathrm{of}\;\mathrm{negative}\;\mathrm{control}}\;\times 100$$

#### 3.6.12. In Vitro Cytotoxicity via MTT Assay

An MTT assay was used to determine the biocompatibility of the formulations. Macrophages RAW 264.7 cells were seeded in polystyrene 96=well plates at a density of 5000 cells/well supplemented with DMEM media along with 10% (*v*/*v*) FBS and 1% antibiotics, followed by incubation in CO_2_ incubator at 37 °C. Mucoadhesive tablets were diluted and re-suspended in DMEM in the concentration ranges of 3.125, 6.25, 12.5, 25, 50 and 100 µg/mL, followed by incubation in CO_2_ incubator at 37 ± 0.5 °C for 24 h. Furthermore, the culture media replacement with fresh media and 500 µg/mL MTT/PBS solution following 4 h incubation. Violet-colored formazan crystals were formed by the interaction of MTT and viable cells. DMSO (100 µL) was added to each well to dissolve formazan crystals, and absorbance at 540 nm was measured via multiplate reader [53]. (Perkin Elmer, Waltham, MA, USA). The cell viability was calculated as follows (Equation (7)):

(7) $$\%\;\mathrm{cell}\;\mathrm{viability}=\frac{\mathrm{Absorbance}\;\left(\mathrm{sample}\right)-\mathrm{Absorbance}\;\left(\mathrm{blank}\right)}{\mathrm{Absorbance}\;\left(\mathrm{control}\right)-\mathrm{Absorbance}\;\left(\mathrm{blank}\right)}$$

#### 3.6.13. In Vivo Histopathological Analysis

Concisely, two groups of rabbits (*n* = 3) were kept with free access to food and water in a suitable environment. Then, the left maxillary cleft of the rabbit lip was fixed and observed on a daily basis. Afterwards, the controlled and optimized R4 mucosal tissues were removed, followed by fixation in the buffered formalin solution at ambient temperature for 4 h. Furthermore, all the extracted tissues were embedded in the paraffin for the sectioning of tissues in various directions. The extracted sections were then stained with hematoxylin and eosin (H&E) for further microscopic evaluation via imaging.

#### 3.6.14. Stability Study

A stability study was conducted only on the optimized formulation with the conditions set according to International Council for Harmonization (ICH) guidelines. Briefly, tablets were stored under accelerated stability conditions after being packed in aluminum foil at a temperature and relative humidity (RH) of 40 ± 2 °C and 70 ± 5%, respectively, for six months [11,54]. For each specified interval, 10 tablets were removed from the chamber and crushed into fine powder using a mortar and pestle. Then, the weight equivalent to 650 mg was dissolved in 900 mL of mobile phase for the quantitative estimation of MET and SIT for HPLC analysis. Similarly, the MS and ET properties of the tablets were also evaluated. 

#### 3.6.15. Statistical Analysis

It was applied to the optimized formulation. After exposing to stability conditions, the release profile of the dosage form was re-evaluated and subjected to similarity (f2) and dissimilarity (f1) factor analyses by being compared to the in vitro release of drugs in the optimized formulation before stability test exposure. Moreover, a sample paired *t*-test was applied to determine whether the significant difference between the release profiles of the drugs before and after stability existed or not. For the paired Student’s *t*-test, a *p*-value of less than 0.05 was considered as the level of statistical significance [11]. 

## 4. Conclusions

The application of buccal mucoadhesive drug delivery carriers have opened new avenues of advancement in the management of DM, bypassing the various limitations of conventional delivery systems. Bioinspired polymeric blends have shown encouraging results in the management of systemic diseases. We found that the buccal mucoadhesive delivery of CP/PVP- based mucoadhesive tablets for managing DM can be an attractive option to deliver drugs. Moreover, the optimized formulation showed a sustained drug release by following the Korsmeyer–Peppas model and the first-order release model, corresponding to SIT and MET. The optimized formulation was also found to be hemocompatible, biocompatible and stabilized. The exorbitant simultaneous drug loading can produce better outcomes in the management of DM, ensuring sufficient pharmaceutical stability.

## Figures and Tables

**Table 1:** Average weight, thickness and diameter of the formulated tablets.

Code	Average Weight	Diameter	Thickness
	mg ± SD	mm ± SD	mm ± SD
R1	649.9 ± 2.59	12.12 ± 0.02	5.67 ± 0.05
R2	652.4 ± 2.19	12.13 ± 0.005	5.66 ± 0.12
R3	650.3 ± 2.61	12.11 ± 0.04	5.67 ± 0.15
R4	650.8 ± 1.69	12.13 ± 0.02	5.63 ± 0.09
R5	651.1± 2.33	12.13 ± 0.04	5.66 ± 0.05
R6	649.1 ± 3.18	12.15 ± 0.01	5.69 ± 0.11
R7	645.4 ± 2.34	12.12 ± 0.06	5.63 ± 0.20
R8	651.0 ± 3.23	12.15 ± 0.01	5.67 ± 0.08
R9	648.2 ± 2.18	12.13 ± 0.07	5.66 ± 0.03
R10	653.1 ± 2.76	12.12 ± 0.01	5.66 ± 0.46
R11	647.9 ± 3.15	12.12 ± 0.01	5.67 ± 0.12
R12	650.0 ± 1.97	12.13 ± 0.01	5.65 ± 0.09
R13	650.7 ± 2.50	12.11 ± 0.01	5.66 ± 0.04
R14	650.1 ± 2.85	12.13 ± 0.01	5.67 ± 0.10
R15	651.6 ± 2.13	12.12 ± 0.01	5.66 ± 0.15

**Table 2:** Physicochemical characterization of the buccal formulations.

Code	Content Uniformity		pH	ME%
	MET% ± SD	SIT% ± SD		
R1	98.09 ± 1.56	100.25 ± 1.10	7.11	73.08
R2	100.02 ± 0.75	99.59 ± 1.17	6.80	19.44
R3	102.40 ± 0.95	99.98 ± 1.53	6.38	39.63
R4	101.29 ± 1.66	100.53 ± 0.32	6.20	18.87
R5	99.53 ± 1.11	101.76 ± 0.95	5.43	24.5
R6	99.11 ± 1.39	100.92 ± 1.23	5.94	62.31
R7	99.74 ± 1.56	99.49 ± 1.77	6.37	70.22
R8	100.07 ± 1.05	98.71 ± 1.20	6.31	39.54
R9	99.20 ± 1.63	97.13 ± 0.78	5.91	90.02
R10	100.36 ± 0.80	100.90 ± 0.75	5.27	40.22
R11	98.77 ± 1.36	101.73 ± 1.36	6.19	20.64
R12	99.70 ± 1.18	102.71 ± 01.42	5.83	13.69
R13	97.08 ± 1.29	98.33 ± 1.22	5.09	18.36
R14	97.37 ± 1.17	99.82 ± 1.41	4.89	13.87
R15	98.85 ± 0.64	99.75 ± 1.08	4.74	34.15

**Table 3:** Ex vivo permeability of the optimized formulation (R4).

Parameters	MET	SIT
Jss (mg/cm^2^.h)	7.359	13.853
Kp (cm/h)	1.47 × 10^−5^	2.77 × 10^−4^

**Table 4:** In vitro release kinetic analysis of MET and SIT from the optimized formulation (R4).

Model	MET r^2^ (*n*)	SIT r^2^ (*n*)
Zero-order model	0.9269	0.128
First-order model	0.9471	0.9765
Higuchi model	0.7433	0.8570
Korsmeyer–Peppas model	0.9633 (0.321)	0.8813 (0.419)
Hixson–Crowell model	0.8582	0.9747

**Table 5:** Stability study of the optimized formulation R4.

Time	Appearance	ET	MS	Contents % ± SD	
(Months)		h ± SD	g ± SD	SIT	MET
1	White to off-white	7.46 ± 3.76	24.54 ± 2.08	99.25 ± 0.54	99.20 ± 1.44
3	White to off-white	7.11 ± 2.19	23.68 ± 2.76	99.37 ± 1.01	98.59 ± 1.52
6	White to off-white	7.78 ± 3.33	26.37 ± 1.32	98.04 ± 1.23	98.31 ± 1.11

**Table 6:** Release profile comparison after stability conditions of the optimized formulation (R4).

Parameter	MET	SIT
Similarity factor (f2)	67.47	62.35
Dissimilarity factor (f1)	5.88	7.17

**Table 7:** Statistical analysis of the optimized formulation (R4) exposed to stability testing.

Before–After Stability	Mean	Standard Deviation	Standard Error Mean	95% Confidence Interval of the Difference		*t*-Value	df	Significance (2-Tailed)
				Lower	Upper			
MET	3.57	2.73	1.11	0.697	6.445	3.194	5	0.21
SIT	3.25	4.95	2.02	−1.947	8.457	1.608	5	0.346

**Table 8:** Percentage composition (%, *w*/*w*) of polymers to formulate the mucoadhesive buccal tablet.

Code	CP	PVP	AG
R1	2	12	-
R2	4	10	-
R3	7	7	-
R4	10	4	-
R5	12	2	-
R6	-	12	2
R7	-	10	4
R8	-	7	7
R9	-	4	10
R10	-	2	12
R11	2	-	12
R12	4	-	10
R13	7	-	7
R14	10	-	4
R15	12	-	2

## Data Availability

The authors confirm that data is contained within the article.
