# Correction: Shakir et al. Exorbitant Drug Loading of Metformin and Sitagliptin in Mucoadhesive Buccal Tablet: In Vitro and In Vivo Characterization in Healthy Volunteers. *Pharmaceuticals* 2022, *15*, 686

**DOI:** 10.3390/ph17050556

**Published:** 2024-04-26

**Authors:** Rouheena Shakir, Sana Hanif, Ahmad Salawi, Rabia Arshad, Rai Muhammad Sarfraz, Muhammad Irfan, Syed Atif Raza, Kashif Barkat, Fahad Y. Sabei, Yosif Almoshari, Meshal Alshamrani, Muhammad Ali Syed

**Affiliations:** 1 Department of Pharmaceutics, Faculty of Pharmacy, The University of Lahore, Lahore 54000, Pakistan; rshak123@hotmail.com (R.S.); rabia.arshad@bs.qau.edu.pk (R.A.); kashif.barkat@pharm.uol.edu.pk (K.B.); 2 College of Pharmacy, University of Sargodha, Sargodha 40100, Pakistan; sarfrazrai85@yahoo.com; 3 Department of Pharmaceutics, College of Pharmacy, Jazan University, Jazan 45142, Saudi Arabia; asalawi@jazanu.edu.sa (A.S.); fsabei@jazanu.edu.sa (F.Y.S.); yalmoshari@jazanu.edu.sa (Y.A.); malshamrani@jazanu.edu.sa (M.A.); 4 Department of Pharmaceutics, Faculty of Pharmaceutical Sciences, Government College University Faisalabad, Faisalabad 38000, Pakistan; 5 Department of Pharmaceutics, Punjab University College of Pharmacy, University of The Punjab, Lahore 54590, Pakistan; raza.pharmacy@pu.edu.pk

## Error in Figure

In the original publication, a mistake was observed in Figure 5 as published [1]. Unfortunately it was, in fact, overlooked while formulating Figure 5, where the dry picture at 0 h for formulation R7 was pasted in place of R8 in the published file. The corrected version of Figure 5 with the rectified image of R8 at 0 h in Figure 5 appears below. 

The authors state that the scientific conclusions are unaffected. This correction was approved by the Academic Editor. The original publication has also been updated.

## Figures and Tables

**Figure 5:**
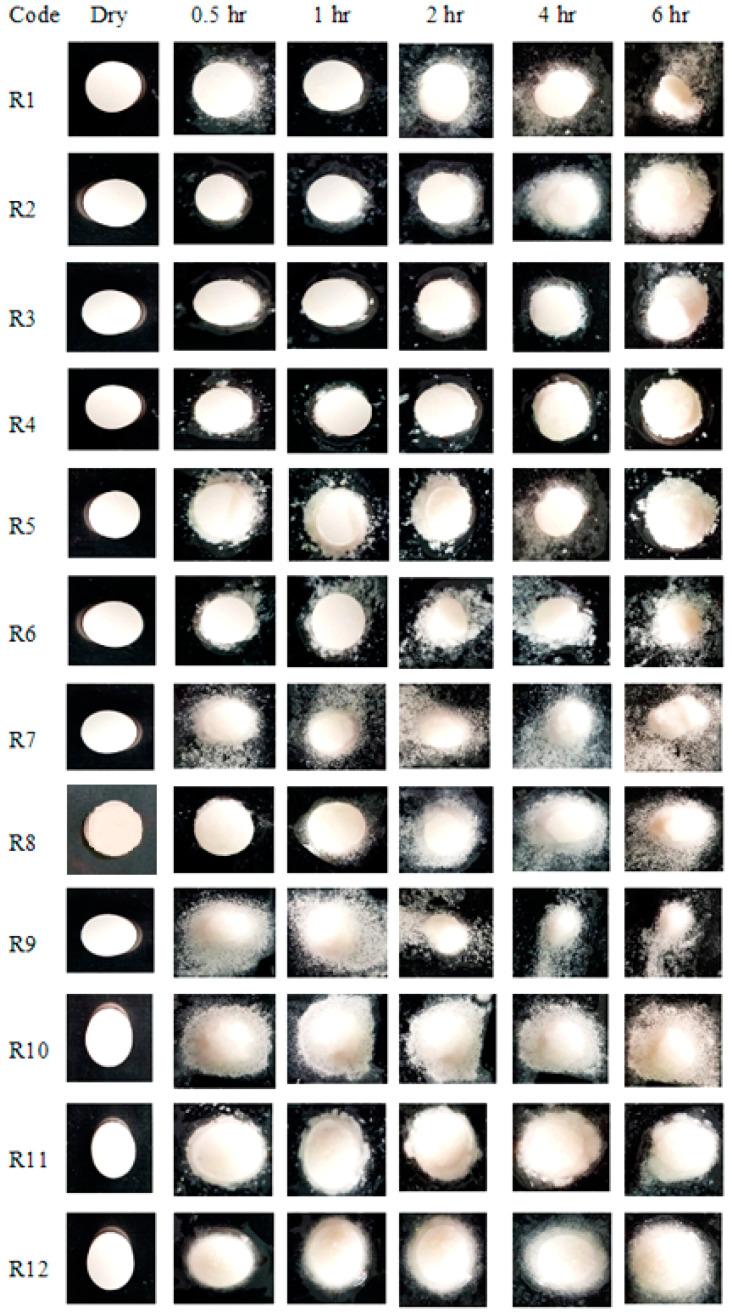
Photographic swelling pattern of the buccal formulations.
